# O.4.4-8 Promoting men’s health through sports clubs: a rapid realist review

**DOI:** 10.1093/eurpub/ckad133.205

**Published:** 2023-09-11

**Authors:** Anne Timm, Aurelie van Hoye, Paul Sharp, Tue Helms Andersen, Peter Krustrup, Louise Hansen, Kenneth Holm Cortsen, Peter Bindslev Iversen, Morten Hulvej Rod, Charlotte Demant Klinker

**Affiliations:** Health Promotion Research, Copenhagen University Hospital – Steno Diabetes Center Copenhagen, Herlev, Denmark; Department of Adaptation, Measurement and Evaluation of Health, University of Lorraine, France; Physical Activity for Health Research Cluster, Health Research Institute, Department of Physical Education and Sport Sciences, University of Limerick, Ireland; School of Nursing and Department of Psychiatry, University of British Columbia, Canada; School of Sport, Exercise and Rehabilitation, University of Technology Sydney, Australia; Department of Education, Steno Diabetes Center Copenhagen, Denmark; Research Unit for Sports and Health Sciences, Department of Sports Science and Clinical Biomechanics, University of Southern Denmark, Odense, Denmark; Danish Institute for Advanced Studies (DIAS), University of Southern Denmark, Odense, Denmark; Department of Education, Steno Diabetes Center Copenhagen, Denmark; Steno Diabetes Center Aarhus, Aarhus University Hospital, Denmark; University College of Northern Denmark, Denmark; Steno Diabetes Center Sjaelland, Holbaek, Denmark; National Institute of Public Health, University of Southern Denmark, Denmark; anne.timm@regionh.dk; Health Promotion Research, Copenhagen University Hospital – Steno Diabetes Center Copenhagen, Herlev, Denmark

## Abstract

**Purpose:**

Traditional health promotion interventions have failed to engage men. Sports clubs offer male-friendly environments that have been identified as an attractive setting for men’s health promotion, but little is known about implementable intervention strategies and subsequent effects. This rapid realist review (RRR) aims to close this gap by assessing context, mechanisms and effects of health promotion interventions targeting men by or in collaboration with sports clubs.

**Methods:**

The RRR methodology was developed in collaboration with a panel of Danish practice and policy representatives and international academics. A systematic literature search was conducted in February 2023 for studies published after 2013 in MEDLINE, Embase, and SportDiscus databases. Included studies: 1) targeted men aged 18+ years, 2) reported health promotion outcomes, and 3) were delivered primarily by or in collaboration with sports clubs. A grey literature search of National Sports Associations and Federations and non-government men’s health organizations was also performed. Dalkin’s conceptualization of mechanisms in realist methodology was used to identify context- mechanism-outcome configurations (CMOc) and develop a program theory. The study follows Cochrane’s guidance for rapid reviews and is registered in Research Registry (ID: reviewregistry1556).

**Results:**

We identified 2.720 studies after duplicates removal. Preliminary analyses indicate that interventions delivered through sports clubs show promise for engaging men and producing favorable health outcomes (e.g., weight loss, increased physical activity and improved social support). The realist synthesis suggests that health interventions delivered through sports clubs (resource mechanism) targeted at men who have high attachment to the sport or club (context), may align with their interests and identities (mechanism), to increase the potential for engagement and health behavior change (outcome). The analysis will be finalised by August.

**Conclusions:**

Our study indicates that men’s health promotion through sports clubs is emergent. While great strides have been made to increase men’s involvement in health promoting activities by delivering interventions through sports clubs, research has predominantly focused on select behaviours (e.g., physical activity) and sub-groups (e.g., sports fans).

**Support/Funding Source:**

This work was funded by the five Steno Diabetes Centers in Denmark, which are partly funded by the Novo Nordisk Foundation.

